# Using health belief model to assess the determinants of HIV/AIDS prevention behavior among university students in Central, Tanzania: A cross-sectional study

**DOI:** 10.1371/journal.pgph.0004305

**Published:** 2025-02-26

**Authors:** Saidi J. Seif, Erick Donard Oguma, Angelina A Joho

**Affiliations:** 1 Department of Clinical Nursing, School of Nursing and Public Health, University of Dodoma, Dodoma, Tanzania; 2 Department of Sexual and Reproductive Health, Tanzania Youth Bright Future Organization, Dodoma, Tanzania; No institution, UNITED KINGDOM OF GREAT BRITAIN AND NORTHERN IRELAND

## Abstract

The adolescent and youth population is at high risk of contracting HIV infection. Little is known in Tanzania regarding the application of Health Belief Model (HBM) in identifying the factors that influence HIV prevention behaviors within this group. This study aims to use the Health Belief Model to assess the determinants of HIV/AIDS prevention behavior among University students in central Tanzania. The analytical cross-sectional study design was conducted among undergraduate students at the University of Dodoma and St. John University in Tanzania, from 15th June 2024 to 15th July 2024. The multiple sampling techniques were employed to select 310 study participants. A structured questionnaire adapted from previous studies was used to collect data from study participants. The determinants of HIV/AIDS prevention behaviors were analyzed using bivariable and multivariable binary logistic regression models. The majority of study respondents 248 (80%) had adequate knowledge of HIV/AIDS prevention behaviors. Most of respondents had high perceived barrier 175 (56.5%) and high perceived benefit 194 (62.6%) toward HIV prevention behavior. More than half of the study respondents had negative attitude 172 (55.5%) and low level of engagement in HIV/AIDS prevention behavior 173 (55.8%). The University students with high perceived barriers (aOR = 0.58, 95% CI: 0.350–0.966; p = 0.036) and negative attitudes (aOR = 0.60, 95% CI: 0.362–0.995; p = 0.048) were less likely to engage in HIV/AIDS prevention behavior practice. This study revealed that more than half of university students were poorly engaged in HIV/AIDS prevention behavior practice. This low level of engagement was mostly influenced by high perceived barriers and negative attitudes toward HIV prevention behavior. Putting more efforts into initiatives to educate university students about HIV prevention practices, will help to maximize their awareness and encourage them to participate in HIV/AIDS prevention behavior.

## Introduction

Human Immunodeficiency Virus (HIV) remains a major public health concern around the world. An estimated more than 39 million people living with HIV globally, the burden of HIV infection contributed to 690,000 deaths and 1.7 million new HIV infections in 2019 worldwide [1]. Africa accounts for more than two-thirds of the global population living with HIV, with over 25 million people living with HIV infection [1].

The Sustainable Development Goal, target 3.3 aims to end the epidemic of HIV/AIDS globally by 2030 [2]. This goal will be achieved when the number of AIDS-related deaths and new HIV infections decline by 90% in 2030. So far, the rate of AIDS-related deaths has declined, whereas the rate of new HIV infections is not on track to achieve SDG target 3.3 especially in sub-Saharan Africa where still the rate of HIV new infection is high, with 880,000 people acquiring HIV and 460,000 people dying from HIV-related disease in 2020 [1].

In Tanzania, prevalence of HIV among adults aged 15 years and older is at (4.4%) with women being more affected. In mainland, the prevalence ranged from 1.7% in Kigoma to 12.7% in Njombe. HIV prevalence of more than (9.0%) was reported in three regions: Mbeya, Iringa, and Njombe [3]. Several efforts have been done to end the HIV pandemic in Tanzania. The UNAIDS established the 95–95–95 targets to ensure that by 2025, 95% of all people living with HIV know their HIV status, 95% of all HIV diagnoses receive sustained ART, and 95% of all ART recipients have Viral Load Suppression [4]. However, the current progress shows that 82.7% of HIV-positive persons (aged 15 and older) were aware of their status, 97.9% were taking ART, and 94.3% had Viral Load Suppression [3].

Tanzania is estimated to have over 61.7 million population, more than 60% of the overall population are children and youths less than 24 years old. The adolescent and youth population account for more than 19% of the total population [5]. This population is at high risk of contracting HIV infection which could be explained by their HIV risk behaviors include unprotected sex, drug abuse, age-disparate relationship, multiple sexual partners, and poor HIV testing [6–9]. HIV prevalence among youth population in Tanzania accounted for 1%, while the annual incidence rate accounted (0.17%) [3]. Addressing the burden of HIV in this age group (15–24) years is the bigger step toward achieving the Global Sustainable Developmental Goals Target 3.3.

The Health Belief Model (HBM) plays a crucial role in predicting the likelihood of engaging in a particular health promotion or prevention behavior through six constructs; perceived severity, perceived benefits, perceived barrier, perceived susceptibility, self-efficacy, and cue to action [10]. This model has been used in previous studies to determine the factors that influence engagement in health promotion or prevention behaviors [11–15]. In the perspective of HIV prevention behavior among the youth population, the same model it has been used. Several factors were identified by the previous studies as the predictors of engagement in HIV prevention behaviors, these factors were perceived barriers, perceived susceptibility, perceived severity, and self-efficacy [11–15].

However, in the context of Tanzania, little is known about the application of the health belief model (HBM) to determine factors influencing HIV prevention behavior among the youth population. Therefore, this study aims to address the gap in using the HBM to assess the determinants of HIV/AIDS prevention behavior among university students in central, Tanzania.

## Methods and materials

### Study setting

The analytical cross-sectional study was conducted at the University of Dodoma and St. John University in Dodoma, central Tanzania, from June 15 to July 15, 2024. These universities were purposive selected because they contain a large number of undergraduate students, with a huge diversification of academic programs.

### Study population

The study population consisted of undergraduate university students (both male and female) studying at the University of Dodoma and St. John University of Tanzania.

### Inclusion criteria

All undergraduate students, aged 18 years and above, both male and female from St. John University and the University of Dodoma were included.

### Exclusion criteria

The students who reported being sick and those who reported to have visual impairments and were unable to provide informed consent were excluded.

### Sample size estimation

Kish and Leslie’s formula (1965) was used to calculate the minimum sample size, using a single population proportion formula based on a previous study conducted in Southwest Ethiopia, to assess the prevalence of engagement in HIV/AIDS prevention behavior among University students, which was found to be 74.5% [15].

After using the proportion of (74.5%), the initial sample size was 292 participants. With the addition of a 20% (58) non-response rate, the overall minimum sample size was 350 participants, however, only 310 participants were willing to take part in the study.

### Sampling technique

The study employed multiple sampling techniques to select two universities and study participants. At the first level, a purposive sampling technique was used to select two universities (University of Dodoma and St. John University of Tanzania), due to their status as the primary universities in Dodoma boasting with a large number of undergraduate students, and a diverse array of academic programs. In the second level, a stratified sampling technique was used to stratify students based on their academic programs. Then, a proportionate sampling technique was used to ensure that a sample was representative of the study population from each academic program. Lastly (third level), a systematic sampling method was employed to select 254 students from the University of Dodoma and 56 students from St. John University.

### Data collection method

The self-administered questionnaire used to collect data from study participants, questionnaires were distributed to study participants, and 10–15 minutes were required to complete the questionnaire. Data were collected from students in their respective hostels. Two research assistants were involved in data collection under the guidance and supervision of the principal investigator. Before data collection, a one-week orientation training was conducted to familiarize them with data collection tools, research objectives, procedures, handling of data, and ethical principles.

### Data collection tools

A structured questionnaire adapted from previous studies used to collect data [16–19]. The tool was first structured in English and then translated into Swahili version which was more convenient to the study participants using the back translation approach. The questionnaire was comprised of eight sections with a total of 65 items. The first section comprised 11 items with multiple responses for assessing student’s demographic information. The second section comprised 20 items with a 5-point Likert scale to assess students’ perception toward HIV/AIDS prevention behavior.

The third section comprised 4 items with a 5-point Likert scale for assessing students’ cue to action. The fifth section comprised 5 items with a 5-point Likert scale for assessing students’ self-efficacy. Sixth section comprised 6 items with a 5-point Likert scale for assessing students’ attitudes toward HIV/AIDS prevention behaviors. The seventh section comprised 10 items with binary responses for assessing students’ knowledge of HIV/AIDS prevention behaviors. The last section comprised 9 items with binary responses for assessing students’ HIV/AIDS prevention behavior.

### Validity and reliability

#### Validity.

The validity was ensured by multiple approaches, to ensure content validity, the existing literature was used to formulate the questionnaire and then shared with a panel of two research experts from the University of Dodoma, School of Nursing and Public Health to confirm if all contents and constructs were covered. Whereby 2 items from the knowledge section were removed because were not qualified to measure knowledge, and 1 item from the section on HIV prevention behavior was also removed since appeared to be duplicated. Moreover, to ensure the external validity, the systematic sampling was used to obtain a representative sample.

#### Reliability.

To ensure reliability, a questionnaire was pre-tested using 10% of sample size, to identify areas of confusion and ambiguous items. To minimize assessment variability, two research assistants were given additional training to ensure that, they consistently understood the items and scoring system. Moreover, the internal consistency of the questionnaire was measured, Cronbach’s alpha was (0.77) for the entire questionnaire, (0.79) for HBM constructs, (0.73) for attitude, (0.76) for knowledge, and (0.77) for HIV/AIDS prevention behavior.

### Study variables

The social demographic factors, knowledge, attitude, and constructs of health belief model (perceived susceptibility, perceived severity, perceived benefit, perceived barrier, cue to action, and self-efficacy) were the independent variables, while HIV/AIDS prevention behavior was the dependent variable.

### Variable measurements

#### 1. Health belief model constructs

##### Perceived susceptibility.

The perceived susceptibility was measured using 5 item questionnaire with 5 point Likert scale 1 = Strongly agree, 2 = Agree, 3 = Neutral, 4 = Disagree, and 5 = Strongly disagree, which gives a total of 25 points. The mean score was computed, those who were below the mean were termed as having low perceived susceptibility while those who were above the mean were termed as having high perceived susceptibility [15].

##### Perceived severity.

The perceived severity was measured using 5 item questionnaire with 5 point Likert scale 1 = Strongly agree, 2 = Agree, 3 = Neutral, 4 = Disagree, and 5 = Strongly disagree, which gives a total of 25 points. The mean score was computed, those who scored below the mean were termed as having low perceived severity and those who scored above were termed as having high perceived severity [16].

##### Perceived benefit.

The perceived benefit was assessed using a 5-item questionnaire that utilized a 5-point Likert scale: 1 = Strongly agree, 2 = Agree, 3 = Neutral, 4 = Disagree, and 5 = Strongly disagree, yielding a maximum score of 25 points. The mean score was computed, respondents who scored below the mean were classified as having low perceived benefit, while those scoring above the mean were classified as having high perceived benefit [15].

##### Perceived barrier.

The perceived barrier was measured using 8 item questionnaire with 5 point Likert scale: 1 = Strongly agree, 2 = Agree, 3 = Neutral, 4 = Disagree, and 5 = Strongly disagree, which gives a total of 40 points. The mean score was computed, respondents who scored below the mean were termed as having a low perceived barrier while those who were above the mean were termed as having a high perceived barrier [16].

##### Cue to action.

Cue to action was measured using 4-item questionnaire with a 5-point point Likert scale: 1 = Strongly agree, 2 = Agree, 3 = Neutral, 4 = Disagree, and 5 = Strongly disagree, which gives a total of 20 points. The mean score was computed, those who scored below the mean were termed having low cue to action while those who were above the mean were termed having high cue to action [20].

##### Self-efficacy.

This determines individual beliefs on his or her capacity to perform a particular HIV preventive behavior. The self-efficacy was measured using 6 item questionnaire with 5 point Likert scale 1 = Strongly agree, 2 = Agree, 3 = Neutral, 4 = Disagree, and 5 = Strongly disagree, which gives a total of 30 points. The mean score was computed, those who were below the mean were termed having low self-efficacy while those who were above the mean were termed having high self-efficacy [14].

#### 2. Attitudes towards HIV/AIDS prevention behavior.

Attitudes toward HIV/AIDS prevention behavior were measured using a 6-item questionnaire employing a 5-point Likert scale: 1 = Strongly Agree, 2 = Agree, 3 = Neutral, 4 = Disagree, and 5 = Strongly Disagree, allowing for a total score of 30 points. The mean score was computed, respondents who scored below the mean were classified as having a negative attitude, and those who scored above were classified as having a positive attitude [21].

#### 3. Knowledge regarding HIV/AIDS prevention.

Knowledge regarding HIV/AIDS prevention behavior was assessed using a 10-item questionnaire with binary responses (Yes or No). Each correct response was scored as 1 point, while incorrect responses received 0 points, resulting in a total possible score of 10. A score of 7 points was used as the cutoff point, those who scored above the cutoff point were considered to have adequate knowledge, while those who scored below were termed to have inadequate knowledge [21].

#### 4. HIV/AIDS prevention behavior practice

HIV prevention behavior was assessed using a 9-item questionnaire with multiple response options. These options were consolidated into two categories: “engaged” and “not engaged,” resulting in a maximum score of 18 points (with 2 points awarded for “engaged” and 1 for “not engaged”). The score of 13 points was used as a cutoff point, those who scored above the cutoff point were considered to have high level of engagement in HIV prevention behaviors, while those who scored below were considered to have a low level of engagement [18].

### Data analysis

The data analysis was done using IBM SPSS version 27 software, several steps used to accomplish the analysis process. Respondents’ demographics, perceptions, cue to action, self-efficacy, knowledge, attitude, and HIV prevention behaviors were analyzed using descriptive analysis and reported in frequencies and percentages. The determinants of HIV/AIDS prevention behavior were analyzed using bivariable and multivariable binary logistic regression models. In a multivariable binary logistic regression model, the final results were presented using Adjusted Odd’s ratio (aOR), Confidence Interval (95% CI), and p-value < 0.05.

### Ethical consideration

The ethical approval to conduct this study was granted by the Institutional Research Review Committee (IRREC) of the University of Dodoma on 14^th^ June, 2024 with Ref, No MA.84/261/75/153. Before data collection informed oral and written consent were obtained from study participants. Participants were free to withdraw from the study anytime they felt to do so. Participant’s values, dignity, and integrity were safeguarded. The coding method and alienation of the subject‘s name in the questionnaires were used to ensure confidentiality and personal privacy. Additionally, after the data collection, the participants’ data were secured and protected against access from unauthorized persons.

## Results

### Social demographic characteristics of study participants (N = 310)

A total of 310 participants were involved in this study, with a total response rate of 88.6%. The majority 254 (81.9%) of respondents were from the University of Dodoma with only 56 (18.1%) from St. John University of Tanzania. Male were 171 (55.2%) and female 139 (44.8%). The mean age of study respondents was (23.49 ± 3) years, with a minimum and maximum age of 18 and 45 years, respectively. 

From Table 1: more than three-quarters of respondents 237 (76.5%) were in the age group of 18–24 years. Most of the respondents 239 (77.1%) were living on campus, In-terms of educational level, the majority of respondents 267 (86.1%) were pursuing a bachelor’s degree, and most of them 106 (34.2%) were in the second year. About 218 (70.3%) of participants were from non-health related programs. Additionally,, about 216 (69.7%) of students had reported a monthly income of less than 200,000Tsh. More than two-thirds of study respondents 218 (70.3%) were sponsored by Tanzania Higher Education Student Loan Board. Most of their religion was Christian (78.7%) and majority 287 (92.6%) of the participant’s marital status were single.

**Table 1:** Social demographic characteristics of study participants (N = 310).

Study variables	Frequency (n)	Percent (%)
**Age groups**		
18–24 years	237	76.5
25–45 years	73	23.5
**Types of campus**		
In campus	239	77.1
Off campus	71	22.9
**Type of University**		
University of Dodoma	254	81.9
St. John university of Tanzania	56	18.1
**Sex**		
Male	171	55.2
Female	139	44.8
**Educational level**		
Diploma	43	13.9
Degree	267	86.1
**Study program**		
Health programs	92	29.7
Non-health programs	218	70.3
**Academic Year**		
Fist year	63	20.3
Second year	106	34.2
Third year	96	31
Fourth year	45	14.5
**Source of finance**		
Sponsored by Higher Education Student Loan Board	218	70.3
Sponsored by other sources	92	29.7
**Monthly income**		
Less than 200,000	216	69.7
Above 200,000	94	30.3
**Religion**		
Christian	244	78.7
Muslim	66	21.3
**Marital status**		
Single	287	92.6
Married	23	7.4

### Description of health beliefs model constructs

Table 2 revealed that majority of study respondents scored high on the constructs of Health Belief Model related to HIV/AIDS prevention behaviors. Specifically, 175 participants (56.5%) reported high perceived barriers to HIV/AIDS prevention, while 171 (55.2%) exhibited high self-efficacy. Additionally, 168 participants (54.2%) had high perceived susceptibility, and 167 (53.9%) indicated high perceived severity. Furthermore, 170 respondents (54.8%) reported strong cues to action. Notably, 194 students (62.6%) expressed a high perceived benefit regarding HIV prevention behaviors. The distribution of participants’ responses on HBM constructs is illustrated in Table 3.

**Table 2:** Distribution of scores on constructs of Health Beliefs Model.

HBM Constructs	Frequency (n)	Percent (%)
**Perceived susceptibility**		
Low	142	45.8
High	168	54.2
**Perceived severity**		
Low	143	46.1
High	167	53.9
**Perceived benefit**		
Low	116	37.4
High	194	62.6
**Perceived barrier**		
Low	135	43.5
High	175	56.5
**Cue to action**		
Low	140	45.2
High	170	54.8
**Self-efficacy**		
Low	171	55.2
High	139	44.8

**Table 3:** The distribution of participants’ responses on Health Belief Model constructs (N = 310).

S/N	Statements	SD	D	N	A	SA
**A: Percieved susceptibility**						
1.	University students are at risk of getting HIV infections?	24	25	40	111	110
2.	Can someone who look healthy have HIV infections?	20	14	25	110	141
3.	I can get AIDS even if I only have sex with one person	21	18	36	117	118
4.	Having multiple partners is the risk for getting HIV infections	7	7	23	88	185
5.	You can get HIV/AIDS anytime when you have unprotected sex	12	11	17	102	168
**B: Percieved severity**		**SD**	**D**	**N**	**A**	**SA**
1.	HIV/AIDS is a fetal disease?	31	26	42	91	120
2.	Can someone die because of HIV/AIDS	8	11	22	99	170
3.	HIV/AIDS is not curable	14	10	32	90	164
4.	HIV/AIDS reduce our body immunity and make us prone to get other infections	6	8	16	84	196
5.	HIV/AIDS treatment require taking pills daily till the end of your life	9	10	17	87	187
**C: Percieved benefit**		**SD**	**D**	**N**	**A**	**SA**
1.	You will be safe from getting HIV infection when you will be faithful to your partner	13	19	52	114	112
2.	The possibility of getting AIDS can be reduced by not having sex before marriage	25	29	57	112	87
3.	Consistency condom use reduce the risk of getting HIV	6	11	48	132	113
4.	Use of PEP and PREP can reduce the risk of getting HIV	9	21	71	114	95
5.	HIV counselling and testing promote HIV prevention	8	16	30	124	132
**D: Percieved barrier**		**SD**	**D**	**N**	**A**	**SA**
1.	It is costly to use condom consistently	32	77	58	77	66
2.	It is difficult to discuss with your partner about condom use?	30	65	71	81	63
3.	Using a condom seems like an insult to my partner	26	89	69	58	68
4.	Religious believe hindering the use of condom	79	92	75	32	32
5.	It is difficult to abstain from sexual intercourse	51	67	77	62	53
6.	I would not get tested for HIV because I was afraid of losing my partner.	27	66	64	80	73
7.	I would not go to a local clinic to be tested for HIV because everyone would know my status	25	49	65	76	95
8.	It is costfull to seek for HIV testing and counselling	28	39	46	82	115
**E: Cue to Action Towards HIV Preventation**		**SD**	**D**	**N**	**A**	**SA**
1.	Often you heard about HIV prevention methods in your friend family or social media	14	14	34	129	119
2.	You know someome who use condom during sexual intercourse	29	36	71	105	69
3.	Some of the people you know have perform HIV self test and counselling	17	28	64	117	84
4.	Some of the people you know have used PREP or PEP	26	41	85	87	71
**F: Self Efficacy Towards HIV Preventation**		**SD**	**D**	**N**	**A**	**SA**
1.	Do you believe you can perform HIV self test and counselling	21	17	50	112	110
2.	Do you believe you can have only one sexual partner	15	24	51	96	124
3.	Do you believe you can use condom constistently in sexaual intercourse	25	50	68	85	82
4.	I would find it’s not difficult to use condoms if my sexual partners refuse it	30	53	95	74	58
5.	I would find it’s not difficult to use condoms with my steady partner	25	46	98	76	65
6.	I would find it’s not difficult to use condoms when I feel that it reduces pleasure	36	43	83	88	60

**SD = Strongly disagree, D = Disagree, N = Neutral, A = Agree, SA = Strongly agree.**

### Attitudes towards HIV/AIDS prevention behaviors

Table 4 indicated that, most of the study participants 172 (55.5%) had a negative attitude toward HIV/AIDS prevention behaviors. Most of the respondents 126 (40.6%) agreed that, the only way to prevent HIV/AIDS is the use of condom, and about 107 (34.5%) of the respondents agreed that it is hard to have one sexual partner. Additionally, 127 (41%) of respondents agreed that they may lose their sexual partner if they say no to sex before marriage.

**Table 4:** Participants’ responses regarding attitudes toward HIV/AIDS prevention behaviors.

Statement Items	Strong disagree n (%)	Disagree n (%)	Neutral n (%)	Agree n (%)	Strong agree n (%)
1.Condom use is the only way to prevent HIV	38 (12.3)	56 (18.1)	90 (29.0)	84 (27.1)	42 (13.5)
2.HIV cannot prevented by PEP and PREP	44 (14.2)	60 (19.4)	103 (33.2)	71 (22.9)	32 (10.3)
3.It’s hard to have one sexual partner	70 (22.6)	66 (21.3)	67 (21.6)	68 (21.9)	39 (12.6)
4.Condom use creates doubt between sexual partner	50 (16.1)	69 (22.3)	85 (27.4)	68 (21.9)	38 (12.3)
5.I may lose my partner if I say no to sex	67 (21.6)	55 (17.7)	61 (19.7)	67 (21.6)	60 (19.4)

### Knowledge of HIV/AIDS prevention behaviors

The majority of respondents 248 (80%) had adequate knowledge of HIV/AIDS prevention behaviors. The majority showed adequate understanding particularly on the use of condoms as a preventive measure 288 (92.9%), abstaining from sexual intercourse reduces the risk of getting HIV/AIDS infection 282 (91.0%), sharing of sharp utensils increase high risk of getting HIV 295 (95.2%), HIV self-test and counseling promote practicing HIV preventive measure 300 (96.8%), and lastly having one sexual partner help to prevent HIV/AIDS 300 (96.8%). Refer to Table 5.

**Table 5:** Participants’ responses regarding knowledge of HIV/AIDS prevention behaviors.

Statements	Frequency (n)	Percent (%)
1.HIV can be prevented through the correct use of a condom.		
No	22	7.1
Yes	288	92.9
2.Abstaining from sexual intercourse reduces your risk of contracting HIV infection.		
No	28	9.0
Yes	282	91.0
3.HIV self-test and counseling promote practicing HIV preventive measures.		
No	10	3.2
Yes	300	96.8
4.You can get an HIV infection when you share sharp utensils.		
No	15	4.8
Yes	295	95.2
5.PEP is the pill used to prevent HIV infection before exposure.		
No	99	31.9
Yes	211	68.1
6.Correct use of PREP helps to prevent a person from getting HIV infection by 99%.		
No	98	31.6
Yes	212	68.4
7.PREP is the pills used to prevent HIV infection after being exposed.		
No	83	26.8
Yes	227	73.2
8.Having one partner reduce the risk of getting HIV infection?		
No	10	3.2
Yes	300	96.8
9.People are likely to get HIV infection by deep kissing		
No	130	41.9
Yes	180	58.1
Overall knowledge		
Inadequate	62	20
Adequate	248	80

### HIV/AIDS Prevention Behavior

From Table 6: The majority of study participants 173 (55.8%) had a low level of engagement in HIV/AIDS prevention behaviors. More than three-quarters of students 250 (80.6%) had already engaged in sex, and about two-thirds of students 214 (69%) had sexual intercourse in the last 12 months. Among the University students who have engaged in sexual intercourse, only 42 (13.5%) used condoms consistently. About 169 (54.5%) did not test for HIV/AIDS status in the past 3 months, and more than half of the students had one sexual partner 178 (57.4%).

**Table 6:** Participants’ responses regarding HIV/AIDS prevention behavior (N = 310).

Variables	Category	Frequency (n)	Percent (%)
Already had sex	Yes	250	80.6
	No	60	19.4
Had a sex in last 12 month	Yes	214	69.0
	No	96	31.0
Types of sexual partner	Regular	180	58.1
	Casual	67	21.6
	Commercial	8	2.6
	Single	55	17.7
Number of sexual partner	One	178	57.4
	Two	60	19.4
	More than 3	25	8.1
	Single	47	15.2
Used condom in last 12 month	Yes	133	42.9
	No	177	57.1
Frequency of condom use	Consistently	42	13.5
	Sometimes	101	32.6
	Occasionally	52	16.8
	Never	115	37.1
Tested HIV/AIDS	Yes	141	45.5
	No	169	54.5
Shared sharp utensil	Yes	79	25.5
	No	231	74.5

### Determinants of HIV/AIDS prevention behavior among university students

In multivariable binary logistic regression model, the results show that high perceived barriers and negative attitudes were the determinants of HIV/AIDS prevention behaviors among University students. From Table 7: University students with high perceived barriers were less likely to engage in HIV/AIDS prevention behaviors compared to students with low perceived barriers (aOR = 0.58, 95% CI: 0.350–0.966; p = 0.036). Also, University students with negative attitudes towards HIV/AIDS prevention behaviors were less likely to engage in HIV/AIDS prevention behaviors compared to those with positive attitudes (aOR = 0.60, 95% CI: 0.362–0.995; p = 0.048).

**Table 7:** Bivariable and multivariable binary logistic regression analysis showing determinants of HIV/AIDS prevention behavior among University students (N = 310).

Variables	cOR	95% CI		p-value	aOR	95% CI		p-value
		Lower	Upper		Lower	Upper		
**Sex**								
Male	2.06	1.305	3.252	0.002	0.641	0.392	1.046	0.075
Female	(Ref)							
**Age groups**								
18–24 years	1.019	0.601	1.729	0.94	1.079	0.612	1.900	0.793
24–45 years	(Ref)							
**Educational level**								
Diploma	1.385	0.726	2.640	0.323	1.288	0.644	2.575	0.475
Degree	(Ref)							
**Perceived susceptibility**								
Low	1.318	0.84	2.069	0.229	1.153	0.683	1.946	0.595
High	(Ref)							
**Perceived severity**								
Low	1.509	0.961	2.37	0.074	1.278	0.751	2.174	0.366
High	(Ref)							
**Perceived benefit**								
Low	1.232	0.086	1.232	0.378	1.055	0.610	1.823	0.848
High	(Ref)							
**Perceived barrier**								
High	0.439	0.277	0.695	0.000	0.581	0.350	0.966	0.036*
Low	(Ref)							
**Cue to action**								
Low	0.942	0.601	1.478	0.795	0.856	0.515	1.422	0.547
High	(Ref)							
**Self-efficacy**								
Low	1.654	1.048	2.609	0.031	1.454	0.875	2.144	0.148
High	(Ref)							
**Attitude**								
Negative	0.424	0.268	0.672	0.000	0.600	0.362	0.995	0.048*
Positive	(Ref)							
**Knowledge level**								
Inadequate	0.72	0.459	1.129	0.152	1.431	0.754	2.716	0.273
Adequate	(Ref)							

## Discussion

The study aimed to use the Health Belief Model to assess the determinants of HIV/AIDS prevention behavior among University students in Central Tanzania. The results revealed that more than three-quarters of study respondents had adequate knowledge of HIV/AIDS prevention behaviors. These findings were supported by the previous studies conducted in the United States and China, which also indicated the majority of students had an adequate understanding of HIV prevention behaviors [17,22]. 

Most of the students were aware, that HIV can be transmitted through unprotected sex and sharing sharp utensils. Also recognized the importance of abstaining from sexual intercourse and consistent using condoms in preventing HIV infection [17,22]. The similarities in these findings may be influenced by HIV campaigns and mass media education. University students are more likely to be informed about various health topics and to participate in various HIV-related programs. These results highlight the efforts made by different stakeholders to provide education through the implementation of several university-level HIV-based initiatives.

Moreover, the study revealed that more than half of university students had negative attitudes towards HIV prevention behaviors. Mostly agree that they may lose their sexual partner if they say no to sex before marriage, it is hard to have only one sexual partner and it is costful to use condoms. These findings aligned with the study conducted in China, which also showed that the majority of students believe that using condoms is costful [23]. In contrast to the study conducted in Sudan, which revealed that the majority of students have a positive attitude towards abstaining from sexual intercourse before marriage [24]. The discrepancy in these studies may be explained by their variation in religion, study area, and cultural background. According to these findings, it is crucial to keep educating young people to reduce their negative attitudes.

Furthermore, more than half of the participants had a high perceived barrier toward HIV prevention behaviors. This finding was consistent with the study conducted in Albania which revealed that most of the students perceived the use of condoms seem like an insult to their partner [16]. The similarities in these studies may be influenced by the same cultural background, in fact both studies were conducted in Africa, where there is a high negative attitude towards the use of condoms. Moreover, findings revealed that more than half of the participants had a high perceived severity of HIV infection. A similar finding has been reported in the study conducted in Ghana [25]. The reason for similarity may be attributed to the burden of HIV and HIV-related death in Sub saharan Africa [1].

Additionally, the study revealed that the majority of the students had low-level engagement in HIV prevention behaviors. Only 42 (13.5%) students used condoms consistently during sexual intercourse, and more than three-quarters did not test for HIV status in the past 3 months. A similar finding was reported by a study done in Colombia which showed that more than three-quarters of students had never been tested for HIV Sero status in the past 3 months [13]. The reasons for similarities may be influenced by a low level of awareness of HIV prevention behaviors, high HIV perceived barriers, and negative attitudes which might limit their self-efficacy toward HIV prevention behaviors, particularly in testing and using HIV-protecting barriers like condoms. However, the study conducted in Ethiopia and China revealed majority of students engaged in HIV/AIDS prevention behaviors [26,27]. The variation in these findings could be explained by their variation in levels of knowledge, attitude, perception, and even study area.

In this study, high perceived barriers to HIV prevention, such as concerns about cost, stigma, or lack of access to resources, were found to be associated with lower engagement in HIV/AIDS prevention behaviors. When individuals believe that these barriers are insurmountable, they may feel discouraged from seeking information, getting tested, or adopting preventive measures such as using condoms. This creates a cycle in which the perception of difficulty or obstacles reduces progress [28,29].

Therefore, the use of the health belief model is not adequate in explaining factors that influence engagement in HIV prevention behaviors. Further research would be beneficial if may focus on testing a new or another existing theory or model to better account for the effect of HIV/AIDS preventative behavior practices.

## Conclusion

This study revealed more than half of university students are poorly engaged in HIV/AIDS prevention behavior practice. This low level of engagement is mostly influenced by high perceived barriers and negative attitudes regarding HIV prevention behaviors. Putting more efforts into initiatives to educate university students about HIV prevention practices, will help to maximize their awareness and encourage them to participate in HIV/AIDS prevention behavior practices. In the context of this study, HBM better explained how perceived barriers have a significant impact on HIV/AIDS prevention behavior practice. However, the remaining constructs were unable to explain the effect on HIV/AIDS prevention behavior practices. Further research would be beneficial if may focus on testing a new or another existing theory or model for better account for the effect on HIV/AIDS preventative behavior practices.

## Limitations

The study included two universities, both located in urban areas, and involved only 310 undergraduate students, which may limit the generalizability of the findings. Consequently, care should be taken while interpreting the results. Future studies should consider using a larger sample size, incorporating both urban and rural universities.

## Supporting information

S1 Data Data template of study participants. (XLTX)
